# Supplementary material for: Role and mechanism of NCAPD3 in promoting malignant behaviors in gastric cancer
Source: Front Pharmacol. 2024 Apr 22;15:1341039. doi: 10.3389/fphar.2024.1341039 (PMC11070777; doi:10.3389/fphar.2024.1341039)
Supplement: Supplementary file 11 [file DataSheet2.ZIP › GSEA/immunologic/my_analysis.Gsea.1599462003817/GSE17974_0H_VS_24H_IN_VITRO_ACT_CD4_TCELL_UP.html]

Details for gene set GSE17974\_0H\_VS\_24H\_IN\_VITRO\_ACT\_CD4\_TCELL\_UP[GSEA]

|  || Dataset | filtered\_dataset.sample\_info.cls#WT\_versus\_NCAPD3\_MUT |
| Phenotype | sample\_info.cls#WT\_versus\_NCAPD3\_MUT |
| Upregulated in class | NCAPD3\_MUT |
| GeneSet | GSE17974\_0H\_VS\_24H\_IN\_VITRO\_ACT\_CD4\_TCELL\_UP |
| Enrichment Score (ES) | -0.63583905 |
| Normalized Enrichment Score (NES) | -3.1616564 |
| Nominal p-value | 0.0 |
| FDR q-value | 0.0 |
| FWER p-Value | 0.0 |
Table: GSEA Results Summary

  

Fig 1: Enrichment plot: GSE17974\_0H\_VS\_24H\_IN\_VITRO\_ACT\_CD4\_TCELL\_UP      
 Profile of the Running ES Score & Positions of GeneSet Members on the Rank Ordered List

  

| SYMBOL | TITLE | RANK IN GENE LIST | RANK METRIC SCORE | RUNNING ES | CORE ENRICHMENT || 1 | 200315 | APOBEC3A | 866 | -0.278 | -0.6048 | No |
| 2 | 4209 | MEF2D | 910 | -0.337 | -0.6122 | Yes |
| 3 | 301 | ANXA1 | 914 | -0.342 | -0.5903 | Yes |
| 4 | 1195 | CLK1 | 920 | -0.349 | -0.5695 | Yes |
| 5 | 9314 | KLF4 | 972 | -0.389 | -0.5790 | Yes |
| 6 | 10365 | KLF2 | 1000 | -0.405 | -0.5700 | Yes |
| 7 | 51646 | YPEL5 | 1014 | -0.413 | -0.5503 | Yes |
| 8 | 23764 | MAFF | 1039 | -0.436 | -0.5371 | Yes |
| 9 | 7378 | UPP1 | 1111 | -0.484 | -0.5543 | Yes |
| 10 | 388403 | YPEL2 | 1161 | -0.527 | -0.5527 | Yes |
| 11 | 4084 | MXD1 | 1167 | -0.535 | -0.5187 | Yes |
| 12 | 7128 | TNFAIP3 | 1198 | -0.573 | -0.5001 | Yes |
| 13 | 2043 | EPHA4 | 1228 | -0.602 | -0.4788 | Yes |
| 14 | 114907 | FBXO32 | 1248 | -0.625 | -0.4486 | Yes |
| 15 | 2355 | FOSL2 | 1262 | -0.648 | -0.4125 | Yes |
| 16 | 7994 | KAT6A | 1279 | -0.666 | -0.3773 | Yes |
| 17 | 202 | AIM1 | 1290 | -0.681 | -0.3367 | Yes |
| 18 | 57561 | ARRDC3 | 1324 | -0.744 | -0.3082 | Yes |
| 19 | 5997 | RGS2 | 1336 | -0.770 | -0.2621 | Yes |
| 20 | 56605 | ERO1B | 1340 | -0.778 | -0.2097 | Yes |
| 21 | 113791 | PIK3IP1 | 1345 | -0.787 | -0.1573 | Yes |
| 22 | 27076 | LYPD3 | 1348 | -0.790 | -0.1033 | Yes |
| 23 | 133746 | JMY | 1350 | -0.793 | -0.0484 | Yes |
| 24 | 51274 | KLF3 | 1406 | -1.296 | 0.0029 | Yes |
Table: GSEA details [plain text format]

  

Fig 2: GSE17974\_0H\_VS\_24H\_IN\_VITRO\_ACT\_CD4\_TCELL\_UP      
 Blue-Pink O' Gram in the Space of the Analyzed GeneSet

  

Fig 3: GSE17974\_0H\_VS\_24H\_IN\_VITRO\_ACT\_CD4\_TCELL\_UP: Random ES distribution      
 Gene set null distribution of ES for **GSE17974\_0H\_VS\_24H\_IN\_VITRO\_ACT\_CD4\_TCELL\_UP**
